# Cutting Performance of YG8 Cemented Carbide Tools with Microcapsule-Filled Surface Microtextures

**DOI:** 10.3390/ma19102052

**Published:** 2026-05-14

**Authors:** Jianchi Zhou, Jiaying Shi, Yuxin Zhao, Peng Liu, Xianglong Meng, Hui Chen

**Affiliations:** 1 School of Mechanical Engineering, Shandong Key Laboratory of CNC Machine Tool Functional Components, Qilu University of Technology (Shandong Academy of Sciences), Jinan 250353, China; 13306191161@163.com (J.Z.); 15194049002@163.com (P.L.); mengxl_me@qlu.edu.cn (X.M.); 2 Shandong Institute of Mechanical Design and Research, Jinan 250031, China; 3 Department of Intelligent Manufacturing, Shandong Labor Vocational and Technical College, Jinan 250353, China; 2003120196@sdlvtc.edu.cn

**Keywords:** microtextures, self-lubricating microcapsules, dry cutting, cemented carbide tools

## Abstract

To improve the dry cutting performance of YG8 cemented carbide tools, CaF_2_/[BMIM]PF_6_@PPSU solid–liquid dual-core microcapsules were incorporated into microtextures on the rake face, thereby constructing a microcapsule–microtexture composite self-lubricating tool system. Cutting experiments were conducted to systematically investigate the effects of microcapsule content and microtexture edge spacing on the cutting performance of the tools. The results indicate that optimal cutting performance is achieved at a microcapsule content of 20 wt.% and an edge spacing of 100 μm. Under these conditions, the tool embedded with dual-core microcapsules exhibited a main cutting force as low as 88.6 N, a cutting temperature of 237.8 °C, a machined surface roughness of 1.08 μm, and an extended cutting distance of 9497 m. Compared with the unlubricated tool, the main cutting force, axial force, and radial force decreased by approximately 40%, 45.6%, and 47.4%, respectively; the cutting temperature decreased by 43.9%, and the surface roughness was reduced by 24.5%. Micromorphological analysis reveals that, under optimal conditions, the TC2 tool effectively mitigates adhesive and delamination wear on both the rake and flank faces. Energy-dispersive spectroscopy (EDS) analysis demonstrates that the rupture of microcapsules releases two core materials, forming a stable solid–liquid biphasic lubricating film that effectively suppresses adhesive and abrasive wear.

## 1. Introduction

As a green manufacturing technology, dry cutting eliminates the use of cutting fluids during machining, thereby effectively reducing processing costs and avoiding environmental pollution [1,2,3,4]. However, under dry cutting conditions, severe plastic deformation and intense friction at the tool–workpiece and tool–chip interfaces readily generate temperatures exceeding 300 °C, accompanied by high contact pressures and strong adhesive interactions [5]. This not only leads to severe adhesive, abrasive, and thermal wear of the tool, significantly shortening tool life, but also induces fluctuations in cutting forces and plastic deformation of the workpiece surface, resulting in increased surface roughness and making it difficult to meet the machining requirements of high-precision components [6,7,8].

Microencapsulation technology involves encapsulating dispersed solid, liquid, or gaseous substances within natural or synthetic polymeric film-forming materials, thereby forming microscopic particles with a core–shell structure; the encapsulated substance is referred to as the core material, while the film-forming material is termed the shell material [9,10]. By encapsulating lubricants as the core material, self-lubricating microcapsules can be fabricated that combine the structural integrity of solids with excellent lubrication performance [11]. During friction, the microcapsule shell ruptures under shear, releasing the encapsulated lubricant, which subsequently forms a lubricating film on the substrate surface, thereby significantly improving the tribological performance of the material [12]. Yingjie Ma et al. [13] prepared polyurethane microcapsules using interfacial polymerization with [BMIm]PF_6_ as the lubricant core, and subsequently incorporated them into an epoxy resin matrix. Tribological tests demonstrated that, compared with neat epoxy resin, the friction coefficient decreased by 72.7%, while the wear rate was reduced by approximately 160-fold.

As an advanced tool surface modification approach, surface microtexturing technology employs precision techniques such as laser processing and micro-grinding to fabricate regular micro-scale features (e.g., dimples and micro-grooves) on the tool surface, thereby achieving multiple optimization effects at the cutting interfaces [14,15,16]. Surface microtextures on cutting tools not only effectively reduce the contact area between the tool and the chip/workpiece, thereby lowering frictional resistance, but also serve as reservoirs and release channels for self-lubricating media, as well as traps for wear debris, thereby mitigating abrasive wear [17]. For example, by fabricating microtextures on the rake face of WC–8Co tools with a width of approximately 35 μm, a depth of approximately 60 μm, a distance of 100 μm from the cutting edge, and a groove spacing of 100 μm, the high-speed dry turning performance of the tool was significantly improved, with tool life increased by 50%, cutting temperature reduced by approximately 13%, a decrease in the main cutting force, and a marked reduction in adhesion on the rake face [18]. Linear and square microtextures were fabricated on tungsten carbide inserts using focused ion beam (FIB) machining, followed by deposition of a MoS_2_ solid lubricant coating via pulsed DC magnetron sputtering. During dry turning of Al 6063 alloy, the cutting forces of the tools with square and linear microtextures were reduced by approximately 30% and 20%, respectively [19]. The aforementioned studies indicate that filling solid lubricants into surface microtextures on cutting tools can effectively reduce cutting forces and frictional wear. However, directly coated solid lubricants are prone to rapid consumption or delamination during cutting, resulting in limited lubrication durability [20]. Microencapsulation technology offers a promising strategy to address this issue: by encapsulating lubricants within surface microtextures on cutting tools, the microcapsules can rupture under friction-induced shear and release the lubricant, thereby enabling long-lasting and stable lubrication. To the best of our knowledge, the combination of self-lubricating microcapsules with surface microtextures on cutting tools has rarely been explored. Previous studies have focused either on the tribological behavior of microcapsule-containing coatings on planar surfaces or on the performance of microtextured tools filled with solid lubricants (e.g., MoS_2_, graphite) or liquid lubricants without encapsulation. The specific strategy of embedding solid–liquid dual-core microcapsules into laser-ablated micro-reservoirs on the rake face, and exploiting the synergistic lubrication effect under dry cutting conditions, has not been reported in the open literature. Nevertheless, we acknowledge that the individual components of this system (microtextures, microcapsules, and solid/liquid lubricants) have been studied separately, and our contribution lies in the integrated design and verification of the hybrid system for dry machining of hardened steel.

By integrating microencapsulation technology with tool surface microtexturing, the contact area between the tool and the chip/workpiece can be reduced and wear debris can be captured to mitigate abrasive wear, while self-lubricating microcapsules can form a continuous and stable lubricating film on the rake face, thereby further reducing interfacial friction and cutting temperature. This synergistic mechanism—comprising structural lubricant storage, medium-assisted lubrication, and debris capture—effectively overcomes the limitations of single microtexturing (which relies solely on structural friction reduction) and standalone microcapsules (which are prone to loss during operation). Based on this concept, CaF_2_/[BMIM]PF_6_@PPSU microcapsules were incorporated into surface microtextures on YG8 cemented carbide tools, and dry cutting experiments were conducted using quenched and tempered 40Cr steel as the workpiece to investigate the cutting performance and lubrication mechanisms. This study provides an effective approach for improving the dry cutting performance of cutting tools.

## 2. Materials and Methods

### 2.1. Materials

The microcapsules used in this study employed polyphenylsulfone (PPSU) as the shell material, 1-butyl-3-methylimidazolium hexafluorophosphate ([BMIM]PF_6_) as the liquid core, and calcium fluoride (CaF_2_) powder (particle size: 1 μm, purity > 99%) as the solid core; all materials were purchased from Aladdin Reagent Co., Ltd. (Shanghai, China). The cutting tools used were YG8 41305N cemented carbide inserts supplied by Qinghe County Dingfeng Cemented Carbide Co., Ltd. (Qinghe County, Hebei Province, China). The workpiece material was quenched and tempered 40Cr steel with a hardness of 36 ± 1 HRC.

### 2.2. Fabrication of Microcapsule-Filled Microtextured YG8 Cemented Carbide Tools

**Figure 1:**
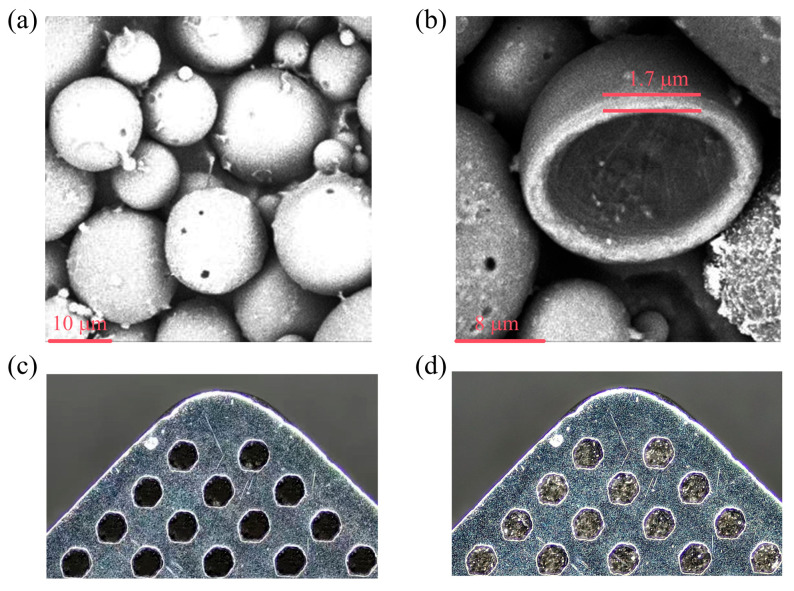
SEM images of [BMIM]PF_6_/CaF_2_@PPSU microcapsules: (**a**) as-prepared microcapsules; (**b**) fractured microcapsules; (**c**) microtextured tool surface before microcapsule filling; (**d**) microtextured tool surface after microcapsule filling (edge-texture distance = 100 μm).

First, the rake face of the tool was ground and polished sequentially using diamond abrasive papers with grits ranging from 500 to 2000, followed by ultrasonic cleaning in acetone for 10 min. Subsequently, surface microtextures were fabricated on the rake face using a Nd:YAG pulsed laser (RFL-P20QQE, Raycus Inc. Wuhan, China). The processing parameters were set as follows: pulse frequency of 40 kHz, power of 50 W, scanning speed of 500 mm s^−1^, and 1000 scanning passes. After processing, the microtextured surface was lightly polished with 1500-grit abrasive paper to remove recast debris. To investigate the effect of dual-core microcapsule-filled microtextures on cutting performance, the microtexture parameters were fixed—based on previous studies [21,22]. The microtextures are circular dimples arranged in a regular array on the rake face, with a diameter of 150 μm, a depth of 100 μm, and edge-texture distance (center-to-center distance) that is varied (50, 100, and 150 μm in the experiments). The areal density of the microtextures, defined as the ratio of the total projected area of the micro-dimples to the total area of the tool surface, was fixed at 20% for the present study. The typical distribution of the microtextures before and after microcapsule filling is shown in Figure 1c,d.

**Figure 2:**
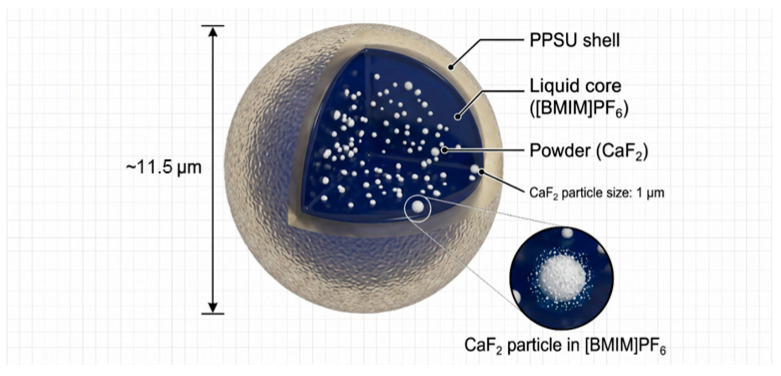
Schematic structure of the dual-core self-lubricating microcapsule.

In this study, polyphenylsulfone (PPSU), featuring high thermal stability and mechanical strength, was used as the shell material, while the ionic liquid [BMIM]PF_6_ and solid lubricant CaF_2_ served as the core materials. The synthesis procedure is briefly described as follows. First, 0.5 g of CaF_2_ powder was mixed with 50 g of [BMIM]PF_6_ and ultrasonically dispersed (220 W, 30 min) to form a homogeneous core suspension. Separately, 1 g of PPSU was dissolved in 40 mL of dichloromethane (DCM), followed by addition of 1.2 g of the core suspension and 0.5 wt.% Span 80 as a surfactant, and the mixture was ultrasonicated for 15 min to obtain the oil phase (Solution A). An aqueous phase (Solution B) was prepared by dissolving 2 wt.% poly (vinyl alcohol) (PVA) in 100 mL deionized water. Under stirring, Solution A was added dropwise into Solution B and then emulsified at 4500 rpm. The resulting emulsion was transferred to a water bath at 35 °C and stirred at 600 rpm for 4 h to completely evaporate DCM. After phase separation, the product was vacuum-filtered, washed repeatedly with ethanol, and finally dried in a vacuum oven at 60 °C for 12 h to obtain dual-core microcapsules. CaF_2_/[BMIM]PF_6_@PPSU dual-core microcapsules were fabricated via a solvent evaporation method, and [BMIM]PF_6_@PPSU single-core microcapsules were also prepared as a control. As shown in Figure 1, the microcapsules exhibit a regular spherical morphology with smooth and dense surfaces, good dispersion, and an average particle size of approximately 11.5 μm, a shell thickness of approximately 1.7 μm. Thermogravimetric analysis indicates a core content of about 41.5 wt.%(liquid [BMIM]PF_6_: ~40 wt.%; solid CaF_2_: ~1.5 wt.%) and thermal stability up to 323 °C. A schematic diagram of the internal structure of the dual-core microcapsules is presented in Figure 2, where CaF_2_ solid particles are dispersed within the [BMIM]PF_6_ liquid core, both encapsulated by a PPSU shell. A photocuring encapsulation technique was employed to achieve stable incorporation of microcapsules within the microtextures. The microcapsules were mixed with a high-temperature-resistant photocurable resin at a predetermined mass ratio to form a composite lubricating slurry, which was uniformly applied onto the microtextured rake face to ensure complete filling of the microtexture dimples. The coated tools were then placed in a vacuum oven at 60 °C and dried under vacuum for 6 h to remove volatile components and achieve pre-curing. Subsequently, curing was carried out using a UV LED light source with a wavelength of 405 nm (irradiation power: 5 W, irradiation distance: 2 cm, irradiation time: 120 s), with 180° double-sided exposure to ensure sufficient crosslinking of the resin. After curing, the rake face was finely polished to remove excess resin, retaining only the microcapsule–resin composite lubricant within the microtexture dimples, thereby obtaining microcapsule-filled microtextured YG8 cemented carbide tools.

It is important to clarify that the “filling content” refers to the mass fraction of microcapsules within the photocurable resin mixture, not the volume percentage of the microtexture being filled. The filling content (*W*) is defined as the mass of microcapsules (*W*_mc_) divided by the sum of the mass of microcapsules and the mass of resin (*W*_re_), calculated as:

(1) $$W\;=\;\frac{W_{mc}}{W_{re}+W_{mc}}\;\times 100\%$$

In this study, five different filling variants were prepared with *W* values of 5, 10, 15, 20 and 25 wt.%.

### 2.3. Performance Evaluation and Characterization

During the cutting experiments, cutting force data were measured in real time using a dynamometer (9129AA, Kistler Group, Winterthur, Switzerland), while the cutting temperature was monitored using a FLIR 315A infrared thermal imaging camera (FLIR Systems, Inc. Wilsonville, OR, USA). The tool wear morphology was initially observed using a VHX-5000 optical microscope (Keyence Corporation, Osaka, Japan), and the surface roughness of the machined workpiece was measured using a white-light vertical scanning interferometer (Contour Elite K, Bruker Corporation, Billerica, MA, USA). Subsequently, a scanning electron microscope (SEM, SUPRA TM55, Carl Zeiss, Oberkochen, Germany) was employed to characterize the wear morphology on both the rake and flank faces, as well as the integrity of the microtextured structures. In addition, an energy-dispersive X-ray spectroscopy system (EDS, IXRF Inc. Austin, TX, USA) was used to analyze the elemental distribution on the rake face after cutting.

### 2.4. Cutting Experiments

In this study, the cutting tool used in this study was a YG8 cemented carbide insert (dimensions: 12.7 mm × 12.7 mm × 5 mm). Cutting tests were conducted on a conventional horizontal lathe (model CDE6140A, Dalian Machine Tool Group, Dalian, China). The tool holder (K-2106FX, KENNAMETAL, PA, USA)) have dimensions of 25 mm × 25 mm × 147.7 mm. The geometric angles of the cutting tool were as follows: rake angle $\gamma_{0}$ = −5°, clearance angle $\alpha_{0}$ = −5°, entering angle $k_{r}$ = 45°, inclination angle $\lambda_{s}$ = 0°, and nose radius $\lambda_{\varepsilon }$ ≈ 0.3 mm. To ensure the reliability and repeatability of the experimental data, each machining test was performed three times (*n* = 3). The results are reported as the mean value ± standard deviation.

Dry cutting experiments were conducted in this study. Based on the cutting parameters for cemented carbide tools machining quenched and tempered steel reported in [4], the feed rate was fixed at *f* = 0.101 mm/rev. A single-factor experimental design was employed to investigate the effects of depth of cut (*a_p_* = 0.2–0.5 mm) and cutting speed (*v* = 100–250 m/min) on the cutting performance of the tool.

The optimal cutting parameters were determined based on the single-factor experiments presented in Table 1 and Table 2. As shown in Table 1, with a fixed cutting speed of 150 m/min, the depth of cut was varied from 0.2 to 0.5 mm. Considering the resulting cutting force, cutting temperature (*T*), and machined surface roughness (*R_a_*), *a_p_* = 0.4 mm was selected because it yielded the lowest surface roughness (*R_a_* = 1.551 μm) while maintaining moderate cutting force (*F_z_* = 164.6 N) and temperature (*T* = 403.5 °C). Subsequently, with the depth of cut fixed at 0.4 mm, the cutting speed was varied from 100 to 250 m/min (Table 2). At *v* = 200 m/min, the surface roughness reached its minimum (*R_a_* = 1.427 μm), and the main cutting force (*F_z_* = 152.7 N) and temperature (*T* = 422.1 °C) remained within acceptable ranges. Higher speeds (*v* = 250 m/min) led to a rebound in surface roughness (*R_a_* = 1.56 μm). Therefore, the combination of *v* = 200 m/min, *a_p_* = 0.4 mm, and *f* = 0.101 mm/rev was identified as optimal and used for all subsequent comparative cutting tests.

## 3. Results and Discussion

### 3.1. Analysis of Cutting Performance

**Figure 3:**
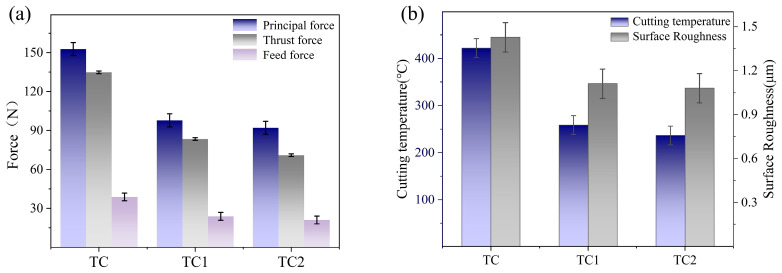
Comparison of cutting performance among three tools: (**a**) cutting force; (**b**) cutting temperature and surface roughness.

Figure 3 illustrates the variations in cutting force, cutting temperature, and machined surface roughness for three types of microtextured tools with a groove spacing of 100 μm. The three tools compared are: (1) the unfilled microtextured tool (designated as TC); (2) the single-core microcapsule-filled tool with [BMIM]PF_6_@PPSU (designated as TC1); and (3) the dual-core microcapsule-filled tool with CaF_2_/[BMIM]PF_6_@PPSU (designated as TC2). The filling content for both TC1 and TC2 tools was 20 wt.%. The results indicate that the TC tool exhibits a main cutting force of 152.7 ± 5 N, a cutting temperature of 422.1 ± 20 °C, and a surface roughness of 1.427 ± 0.1 μm. These results are primarily attributed to severe adhesive wear caused by intense interfacial friction, pronounced plastic deformation, and the accumulation of frictional heat at the tool–chip interface [23]. In contrast, the machining performance of the microcapsule-filled microtextured tools is significantly improved. For the TC1 tool, the main cutting force decreases to 97.8 ± 3 N (a reduction of approximately 35.9%), the cutting temperature drops to 258.7 ± 20 °C (−38.7%), and the surface roughness of the machined workpiece is reduced to 1.11 ± 0.1 μm (−22.4%). The TC2 tool further enhances the cutting performance, with the main cutting force, cutting temperature, and surface roughness reduced to 88.6 ± 3 N, 236.5 ± 20 °C, and 1.08 ± 0.1 μm, respectively, corresponding to reductions of approximately 40%, 43.9%, and 24.5% compared with the TC tool.

**Figure 4:**
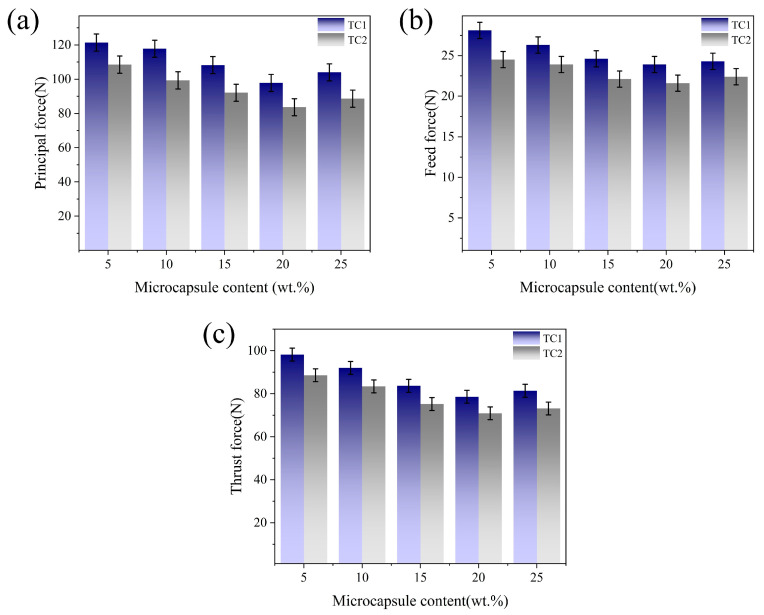
Effect of microcapsule filling content on cutting forces: (**a**) main cutting force; (**b**) axial force; (**c**) radial force.

Figure 4 shows the effect of microcapsule filling content on the cutting force of TC1 and TC2 tools at a edge-texture distance of 100 μm. It can be observed that as the filling content increases from 5 wt.% to 20 wt.%, the cutting force exhibits a continuous decreasing trend. This behavior is attributed to the increased supply of lubricating media, which promotes the formation of a more continuous and dense lubricating film at the tool–chip interface, thereby effectively reducing interfacial shear stress and cutting resistance [24]. However, when the filling content increases to 25 wt.%, all three components of the cutting force show a slight rebound. This phenomenon may be attributed to the agglomeration of excessive microcapsules within the microtextures, resulting in a non-uniform distribution of the lubricating medium and deterioration of the surface structural integrity of the tool. Consequently, local stress concentration is induced, thereby weakening the friction-reducing effect [25]. At the same filling content, the cutting forces of the TC2 tool are consistently lower than those of the TC1 tool.

**Figure 5:**
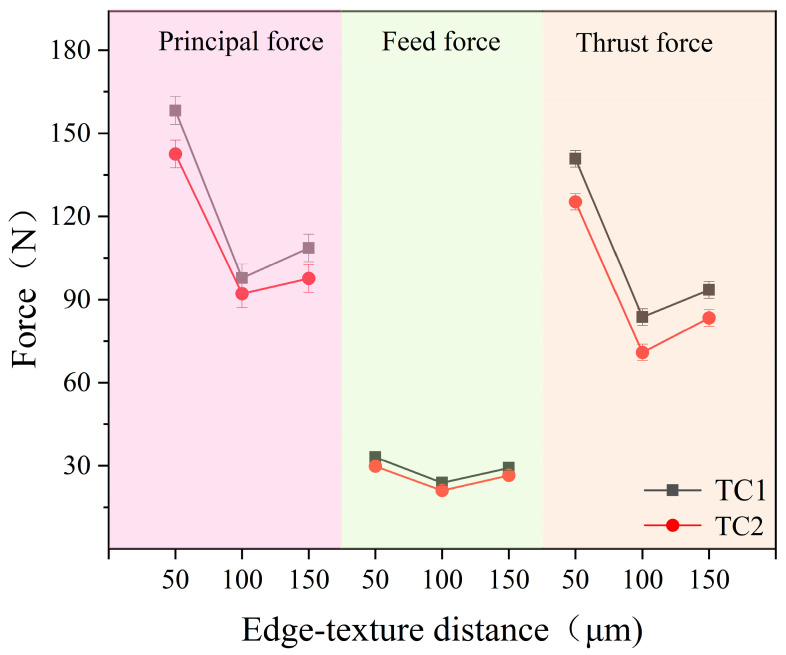
Effect of edge-texture distance on the cutting forces of TC1 and TC2 tools.

Figure 5 illustrates the effect of edge-texture distance on the cutting forces of TC1 and TC2 tools at a microcapsule filling content of 20 wt.%. For both tools, the cutting forces exhibit a decreasing–increasing trend with increasing groove spacing, reaching a minimum at 100 μm. When the groove spacing is 50 μm, the actual tool–chip contact area is excessively large, which not only weakens the structural continuity of the tool substrate and induces stress concentration at the edges of the microtextures, but also accelerates the consumption of the lubricating medium, resulting in relatively high cutting forces. When the spacing increases to 100 μm, the tool–chip contact area is reduced, achieving an optimal balance between lubricant storage/release efficiency and the structural strength of the tool substrate. This ensures sufficient lubricant supply while avoiding stress concentration, thereby effectively reducing interfacial friction and leading to optimal cutting performance. As the spacing further increases to 150 μm, although the contact area continues to decrease, the effect of secondary cutting becomes more pronounced and the supply of lubricating medium becomes insufficient, which aggravates interfacial friction and leads to a rebound in cutting forces [26].

**Figure 6:**
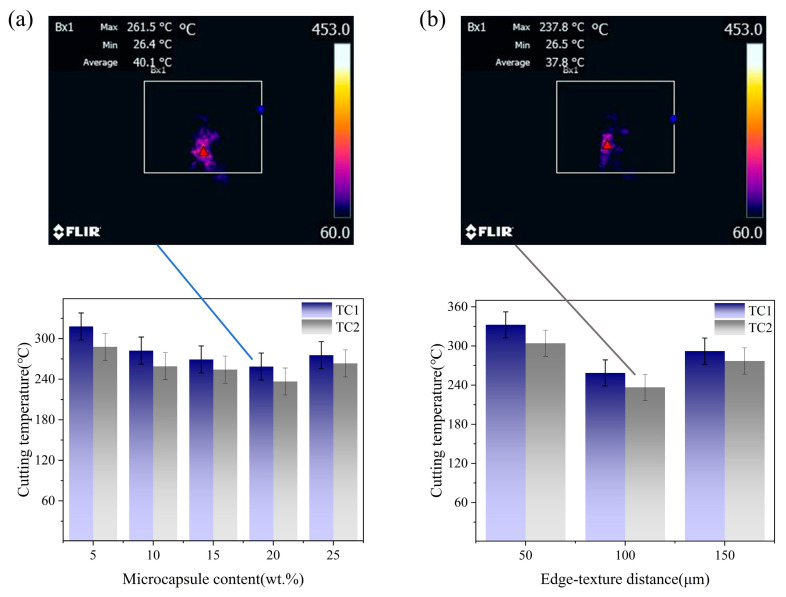
Effect of microcapsule filling content (**a**) and edge-texture distance (**b**) on cutting temperature.

Figure 6 shows the effects of microcapsule filling content (a) and edge-texture distance (b) on the cutting temperature of TC1 and TC2 tools. As illustrated in Figure 6a, the cutting temperature at a filling content of 20 wt.% is significantly lower than that at 5 wt.%. Specifically, for the TC1 tool, the maximum cutting temperature decreases from 317.9 ± 20 °C at 5 wt.% to 261.5 ± 20 °C at 20 wt.%, while for the TC2 tool, it decreases from 287.9 ± 20 °C to 237.8 ± 20 °C. When the filling content is further increased to 25 wt.%, a slight rebound in temperature is observed for both tools, which can be attributed to the agglomeration of excessive microcapsules, leading to non-uniform lubricant distribution and reduced lubrication efficiency. Regarding the effect of groove spacing (Figure 6b), both tools exhibit the lowest cutting temperature at a spacing of 100 μm. At this spacing, an optimal balance between lubricant supply and structural integrity is achieved, resulting in reduced heat generation and enhanced heat dissipation. For the TC1 tool, the cutting temperature decreases from 332.3 ± 20 °C at 50 μm to 261.5 ± 20 °C at 100 μm, while for the TC2 tool, it decreases from 304.1 ± 20 °C to 237.8 ± 20 °C. However, when the spacing is further increased to 150 μm, the reduced proportion of microtextures in the cutting zone leads to insufficient lubrication supply and increased secondary cutting effects, thereby promoting frictional heat accumulation and causing the cutting temperature to rise. Moreover, under identical conditions, TC2 consistently exhibits lower cutting temperatures than TC1, demonstrating the superior thermal regulation capability of the dual-core microcapsule system.

**Figure 7:**
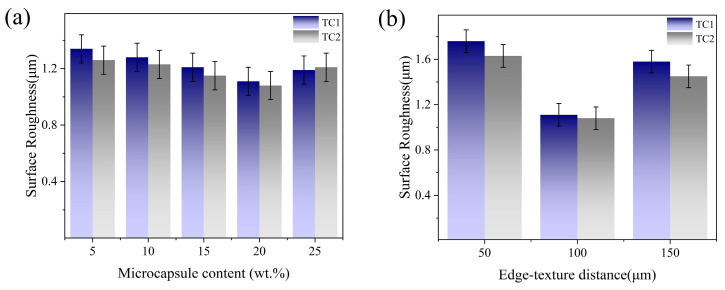
Effects of microcapsule filling content (**a**) and edge-texture distance (**b**) on surface roughness.

Figure 7 illustrates the effects of microcapsule filling content (a) and edge-texture distance (b) on the surface roughness of workpieces machined by TC1 and TC2 tools. As shown in Figure 7a, with the increase in filling content from 5 wt.% to 20 wt.%, a more continuous and stable lubricating film is formed at the cutting interface, leading to a reduction in surface roughness from 1.34 ± 0.1 μm to 1.11 ± 0.1 μm for the TC1 tool and from 1.26 ± 0.1 μm to 1.08 ± 0.1 μm for the TC2 tool. When the filling content further increases to 25 wt.%, agglomeration of microcapsules results in an uneven distribution of lubricant, causing local lubrication failure and inducing cutting micro-vibrations, which deteriorate the surface integrity and lead to a slight increase in surface roughness [27]. As presented in Figure 7b, both tools achieve the lowest surface roughness at a groove spacing of 100 μm. Under identical conditions, the TC2 tool consistently produces a lower surface roughness than the TC1 tool. Compared with the single liquid lubricating film formed by the TC1 tool, the solid–liquid dual-phase lubricating film generated by the TC2 tool exhibits superior load-carrying capacity and stability, which effectively suppresses micro-vibrations during cutting and promotes a smoother interaction between the tool and chip, thereby improving the machined surface quality.

**Figure 8:**
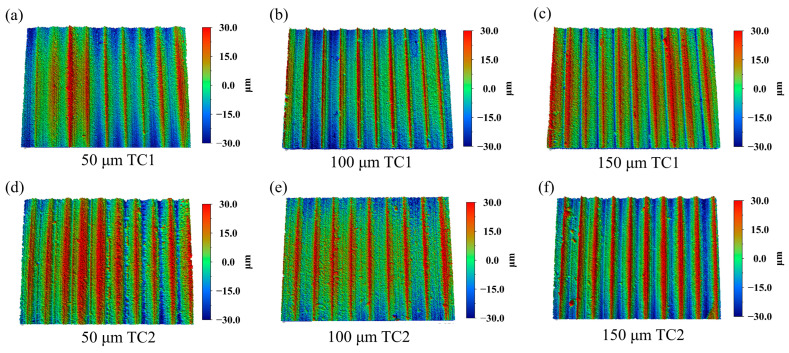
Three−dimensional surface morphologies of workpieces machined by TC1: (**a**) 50 μm, (**b**) 100 μm, (**c**) 150 μm and TC2: (**d**) 50 μm, (**e**) 100 μm, (**f**) 150 μm tools at different edge-texture distances with a filling content of 20 wt.%.

Figure 8 presents the three-dimensional surface morphologies of workpieces machined by TC1 and TC2 tools at different groove spacings with a fixed microcapsule filling content of 20 wt.%. As shown in Figure 8a–c, the surfaces machined by the TC1 tool exhibit pronounced local asperities and depressions under all groove spacing conditions, with more significant height fluctuations observed at a spacing of 50 μm. In contrast, the surfaces produced by the TC2 tool (Figure 8d–f) display a more uniform color distribution, accompanied by a noticeable reduction in both the number and area of extreme peak and valley regions. Notably, at a groove spacing of 100 μm, the surface topography obtained with the TC2 tool demonstrates superior continuity and smoothness compared with that machined by the TC1 tool.

**Figure 9:**
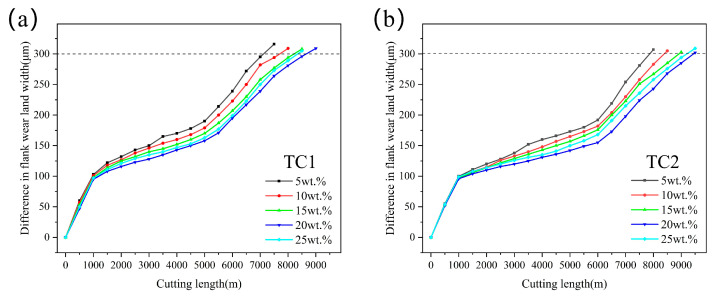
Effects of microcapsule filling content on cutting distance of TC1 (**a**) and TC2 (**b**) tools.

Figure 9 illustrates the effect of microcapsule filling content on the flank wear of TC1 and TC2 tools at a fixed edge-texture distance of 100 μm. For the TC1 tool (Figure 9a), when the flank wear reaches 300 μm, the cutting distance is 7148 m at a filling content of 5 wt.%, which increases to 8667 m at 20 wt.% and then slightly decreases to 8230 m at 25 wt.%. For the TC2 tool (Figure 9b), under the same wear criterion, the maximum cutting distance of 9497 m is achieved at a filling content of 20 wt.%. These results indicate that a filling content of 20 wt.% provides the optimal wear resistance performance for both tools.

**Figure 10:**
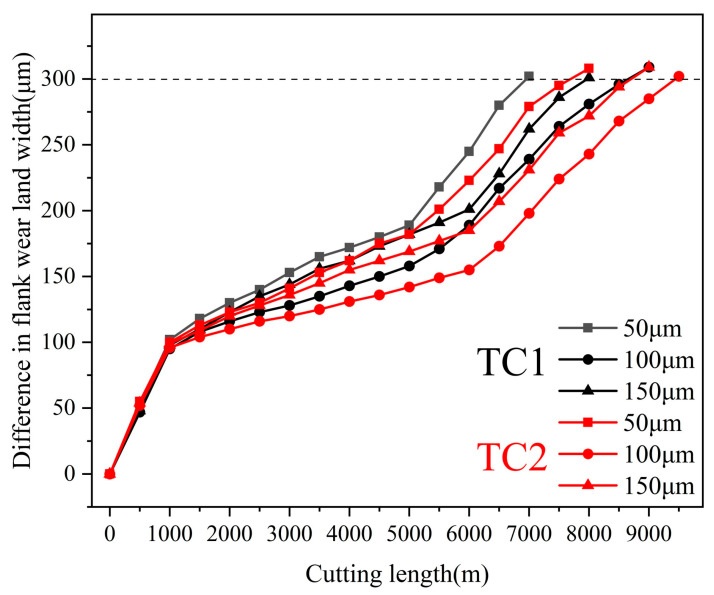
Effects of edge-texture distance on flank wear of tools.

Figure 10 illustrates the effect of edge-texture distance on the flank wear of TC1 and TC2 tools at a fixed microcapsule filling content of 20 wt.%. As shown in Figure 10, the wear curves of the TC2 tool consistently lie below those of the TC1 tool, indicating a lower wear rate. An optimal groove spacing of 100 μm is identified for both tools. Under this condition, the cutting distance of the TC2 tool is approximately 8.7% longer than that of the TC1 tool, demonstrating its superior wear resistance performance.

The single-factor experiments described above have, respectively, revealed the effects of cutting speed, microcapsule filling content, and texture spacing on cutting performance. However, the actual cutting performance of the self-lubricating tool is inherently governed by multiple interactions among mechanical, thermal, and geometric factors. Specifically:

(1) Interaction between cutting speed and microcapsule content: Cutting speed directly affects frictional heat and cutting temperature, thereby regulating the thermal rupture rate of the PPSU shell and the release rate of the lubricant. Under high-speed cutting conditions (e.g., *v* = 250 m/min), a higher microcapsule content (20 wt.%) is essential for maintaining the continuity of the lubricating film and preventing premature depletion of the lubricant. This explains why the 20 wt.% content performed best at *v* = 200 m/min, while at even higher speeds, performance degradation occurred despite the 20 wt.% content.
(2) Interaction between texture spacing and feed rate: The effective spreading and retention of the released [BMIM]PF_6_ liquid at the tool-chip interface depend on the matching between texture spacing and feed rate. A smaller spacing (e.g., 50 μm) at a lower feed rate facilitates the migration and uniform spreading of the liquid core, whereas an excessively large spacing (e.g., 150 μm) under high-feed conditions may lead to localized uneven lubrication, thereby weakening the overall coverage of the lubricating film.

### 3.2. Tool Flank Wear Morphologies

**Figure 11:**
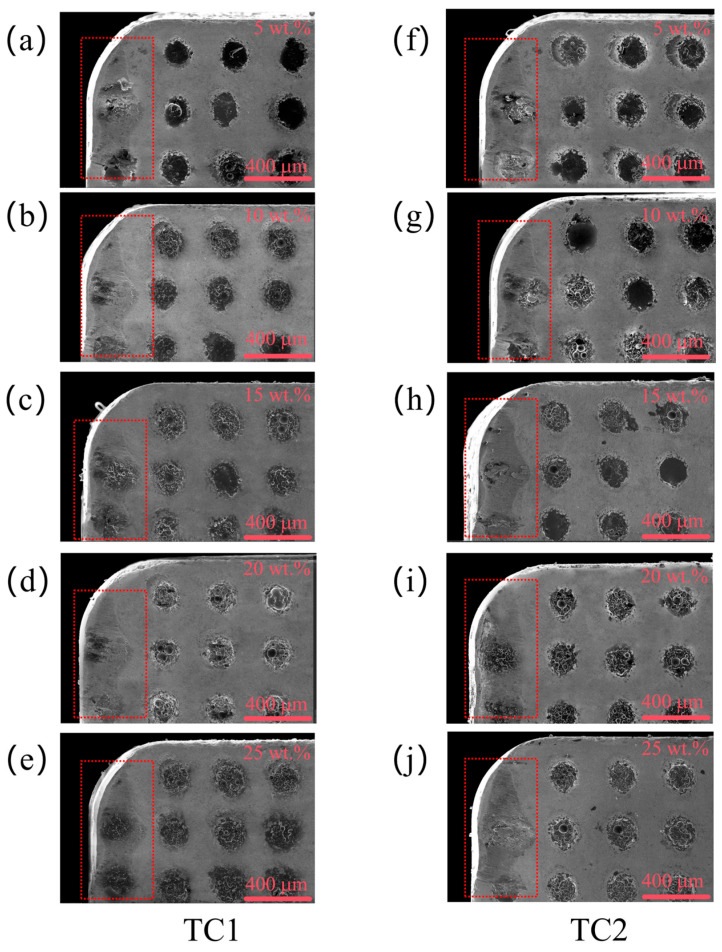
Rake face wear morphologies of TC1 (**a**–**e**) and TC2 (**f**–**j**) tools under different microcapsule filling contents at an edge-texture distance of 100 μm.

Figure 11 presents the rake face wear morphologies of TC1 and TC2 tools under different microcapsule filling contents at a fixed edge-texture distance of 100 μm. At a filling content of 5 wt.%, severe damage is observed on the rake face of the TC1 tool, characterized by deep cavities formed by microcapsule fracture and pronounced scratches on the substrate. When the filling content increases to 10–15 wt.%, the wear condition is partially alleviated; however, localized delamination and non-uniform wear are still evident. At 20 wt.%, the TC1 tool exhibits mainly shallow and relatively uniform surface spalling, with significantly reduced cavity depth and no obvious substrate scratches. In contrast, the TC2 tool demonstrates superior wear resistance under the same conditions, with an almost intact substrate surface. The wear is mainly confined to the superficial layer without propagating into the underlying matrix, indicating a more stable and protective tribological behavior.

**Figure 12:**
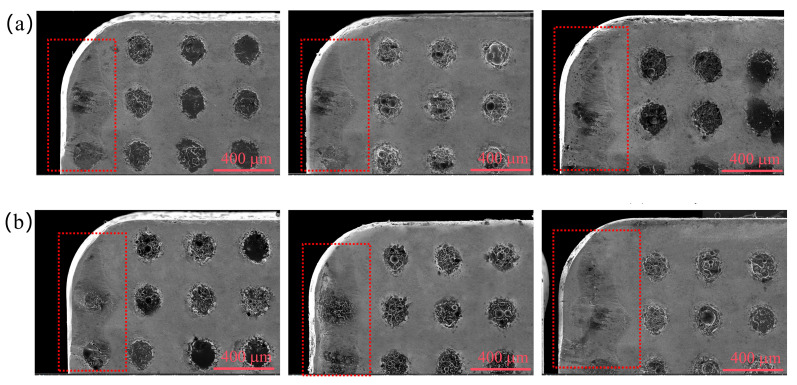
Rake face wear morphologies of TC1 (**a**) and TC2 (**b**) tools under different edge-texture distances at a microcapsule filling content of 20 wt.%.

Figure 12 presents the rake face wear morphologies of TC1 and TC2 tools under different edge-texture distances at a fixed microcapsule filling content of 20 wt.%. At a groove spacing of 50 μm, severe wear is observed on both tools; the TC1 tool exhibits pronounced scratches on the substrate, while the TC2 tool also shows significant surface damage. When the groove spacing increases to 100 μm, the TC1 tool experiences only shallow and relatively uniform surface spalling, whereas the TC2 tool maintains an almost intact substrate with negligible wear. As the groove spacing further increases to 150 μm, the insufficient supply of lubricating medium leads to aggravated wear for both tools. Overall, the TC2 tool consistently exhibits lower wear severity than the TC1 tool across all tested groove spacings, indicating its superior wear resistance under varying lubrication conditions.

**Figure 13:**
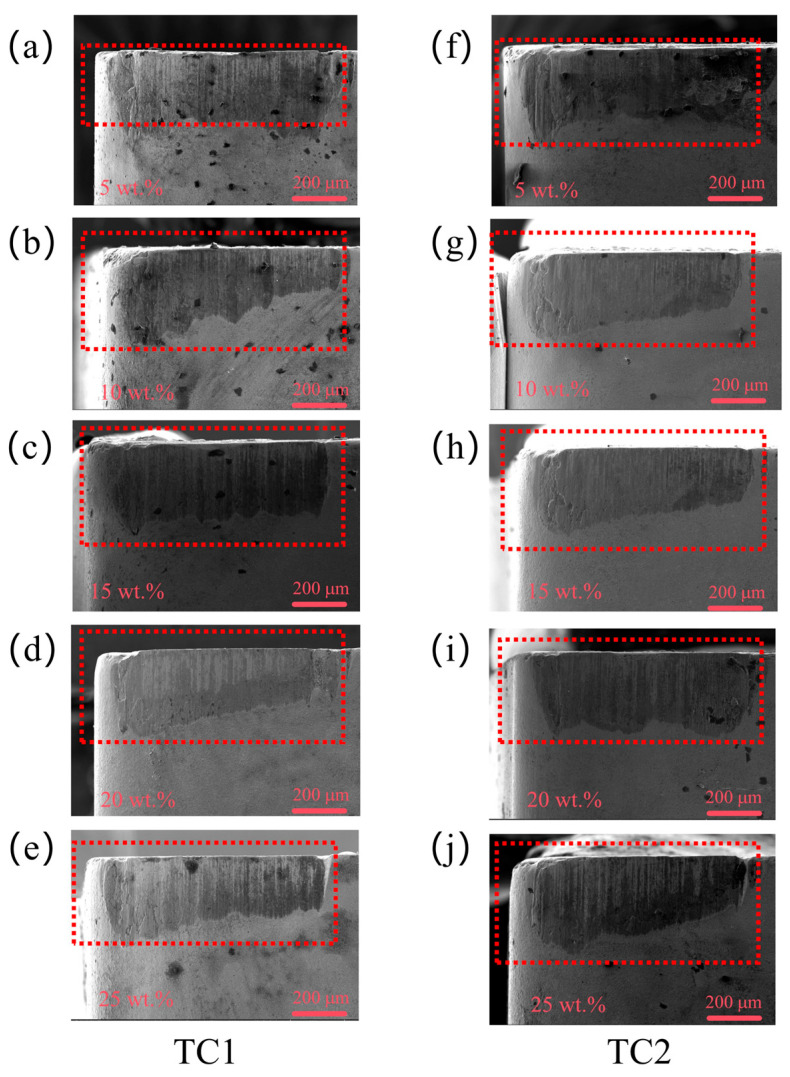
Flank wear morphologies of TC1 (**a**–**e**) and TC2 (**f**–**j**) tools under different microcapsule filling contents at an edge-texture distance of 100 μm.

Figure 13 presents the flank wear morphologies of TC1 and TC2 tools under different microcapsule filling contents at a fixed edge-texture distance of 100 μm. Adhesive wear and abrasive wear are identified as the dominant wear mechanisms during dry cutting processes [28]. As clearly shown in Figure 13, at the optimal filling content of 20 wt.%, the TC2 tool exhibits significantly less flank wear than the TC1 tool. The worn surface of the TC2 tool is relatively smooth and continuous, whereas the TC1 tool still shows residual shallow frictional grooves, indicating a more pronounced wear process.

**Figure 14:**
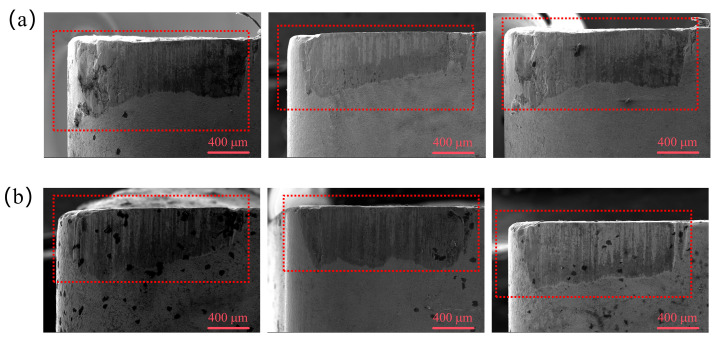
Flank wear morphologies of TC1 (**a**) and TC2 (**b**) tools under different edge-texture distances at a microcapsule filling content of 20 wt.%.

Figure 14 presents the flank wear morphologies of TC1 and TC2 tools under different edge-texture distances at a fixed microcapsule filling content of 20 wt.%. At a groove spacing of 50 μm, severe workpiece material adhesion and spalling pits are observed on the flank face of the TC1 tool. When the groove spacing increases to 100 μm, only a uniform and shallow frictional band remains on the TC1 tool, whereas the TC2 tool exhibits an almost intact surface with negligible wear features. As the groove spacing further increases to 150 μm, both tools show dispersed spalling pits and sporadic adhesive debris on the flank face, indicating aggravated wear due to insufficient lubrication supply.

It is noteworthy that the self-lubricating microtextures on the rake face also provide significant protection for the flank face. This phenomenon is governed by the migratory behavior of the released lubricants. During the semi-finishing process, the [BMIM]PF_6_ liquid core, characterized by its low surface tension and excellent wettability, “creeps” around the tool’s sharp cutting edge to reach the flank-workpiece contact zone.

The size and density of the microtextures dictate the total volume of the lubricant supply. A higher microcapsule content (20 wt.%) ensures a more continuous supply of [BMIM]PF_6_ and CaF_2_ particles. These particles not only lubricate the rake face but also act as a physical barrier on the flank face, reducing the direct mechanical plowing of the 40Cr steel. Furthermore, the reduction in rake-face friction lowers the overall cutting temperature, thereby mitigating diffusion wear and plastic deformation on the flank face.

### 3.3. Cutting Mechanism Analysis

**Figure 15:**
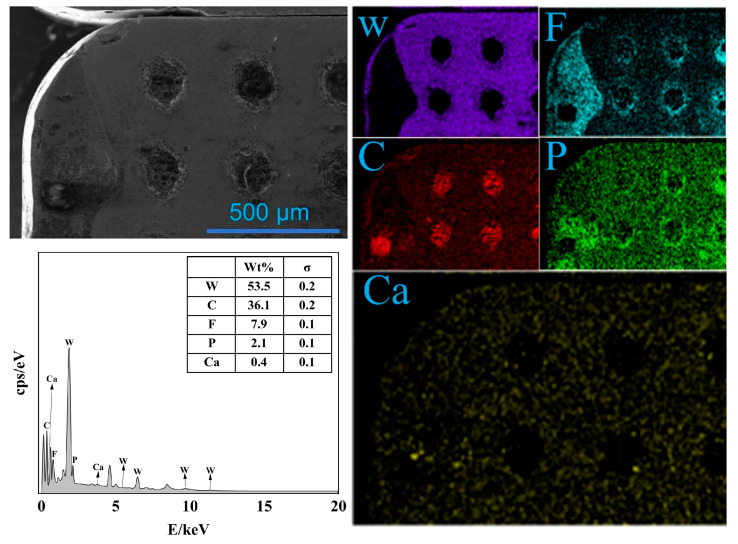
Elemental analysis of the rake face of TC2 at a microcapsule filling content of 20 wt.% and an edge-texture distance of 100 μm.

Figure 15 presents the EDS elemental mapping results of the rake face of the TC2 tool under a microcapsule filling content of 20 wt.% and an edge-texture distance of 100 μm. During the cutting process, the dual-core microcapsules embedded in the TC2 tool fracture and release two types of core materials, forming a continuous solid–liquid composite lubricating film at the tool–chip interface. The EDS results indicate that the rake face is mainly composed of W, C, F, P, and Ca elements. W is primarily associated with the tool substrate and shows a relatively weak signal in regions corresponding to microcapsule cavities. F and P are concentrated in the fractured microcapsule zones, which is consistent with the characteristic elements of [BMIM]PF_6_. The spatial distribution of Ca closely overlaps with F, confirming the presence of CaF_2_. The broadly distributed C signal is attributed to carbonized residues of the PPSU capsule shell as well as adhesion of workpiece material during cutting.

**Figure 16:**
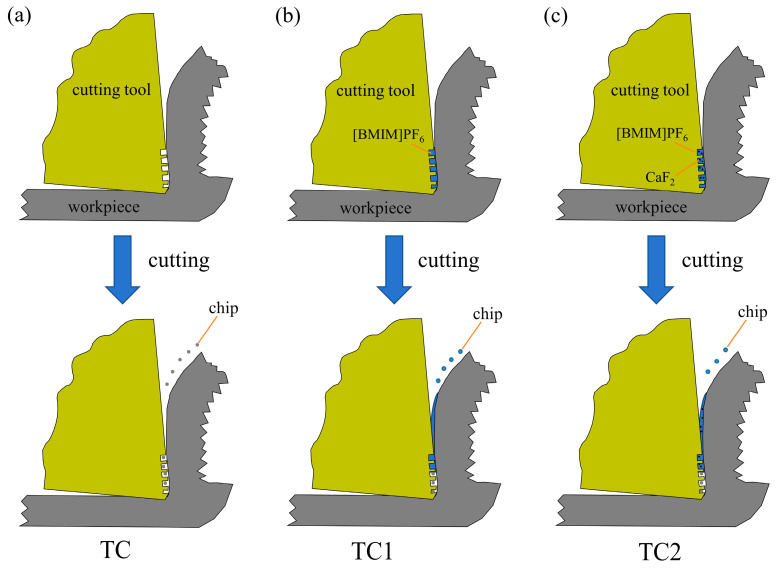
Schematic representation of the synergistic lubrication mechanism on both the rake and flank faces: TC (**a**), TC1 (**b**), and TC2 (**c**).

Figure 16 illustrates the synergistic lubrication mechanism between microcapsule technology and surface microtextures on the cutting tools. The surface microtextures not only reduce the real contact area between the tool and chip, thereby generating a physical friction-reduction effect, but also provide stable micro-reservoirs for microcapsules. Meanwhile, the microcapsules can rupture under mechanical and thermal loads during cutting, releasing the encapsulated lubricants and forming a lubricating film on the tool surface, which significantly improves cutting performance [29]. As shown in Figure 16a, the conventional TC tool relies solely on microtexture pits fabricated on the rake face to achieve friction reduction. On one hand, these microtextures reduce the actual tool–chip contact area and thus decrease interfacial frictional resistance; on the other hand, the microcavities can temporarily trap wear debris, thereby mitigating abrasive wear. For the TC1 tool in Figure 16b, under the combined action of cutting heat and mechanical shear, single-core microcapsules rupture and release [BMIM]PF_6_, forming a liquid lubricating film at the tool–chip interface. This film effectively reduces interfacial shear strength and suppresses adhesive wear. In contrast, the TC2 tool shown in Figure 16c exhibits a superior synergistic lubrication effect. The dual-core microcapsules release both CaF2 and [BMIM]PF_6_ simultaneously. CaF_2_ acts as dispersed solid lubricating particles, forming low-shear-strength discrete sites that reduce direct asperity contact, while [BMIM]PF_6_ fills the interstitial spaces between solid particles, generating a continuous and dense solid–liquid composite lubricating film. The lubricants not only form a tribofilm on the rake face to reduce chip friction but also migrate around the cutting edge to reach the flank face, creating a secondary protective layer at the tool–workpiece interface. Compared with the single liquid-phase lubrication in TC1, this dual-phase lubricating film provides higher load-carrying capacity and stability, thereby leading to significantly improved tribological and cutting performance.

## 4. Conclusions

This study systematically investigates the cutting performance and underlying mechanisms of YG8 cemented carbide micro-textured tools filled with [BMIM]PF_6_@PPSU single-core microcapsules and CaF_2_/[BMIM]PF_6_@PPSU dual-core microcapsules. Through single-factor experiments, the optimal cutting parameters for hardened 40Cr steel were determined as *v* = 200 m/min, *ap* = 0.4 mm, and *f* = 0.101 mm/rev. Based on this, the effects of microcapsule content and edge-texture distance on cutting forces, cutting temperature, surface roughness, and flank wear behavior were further analyzed. The results show that, compared with the TC1 tool filled with [BMIM]PF_6_@PPSU single-core microcapsules, the TC2 tool filled with CaF_2_/[BMIM]PF_6_@PPSU dual-core microcapsules exhibits significantly superior tribological performance due to a solid–liquid synergistic lubrication mechanism. When the filling content is 20 wt.% and the groove spacing is 100 μm, the overall cutting performance reaches its optimum. Compared with the unfilled micro-textured tool TC, the TC2 tool reduces the main cutting force, axial force, and radial force by approximately 40%, 45.6%, and 47.4%, respectively, while cutting temperature decreases by 43.9% and surface roughness is reduced by 24.5%. Micro-morphology analysis indicates that, under optimal conditions, adhesive and delamination wear on both rake and flank faces of the TC2 tool is significantly alleviated. Furthermore, EDS analysis reveals that during cutting, the TC2 tool releases two core materials through microcapsule rupture, forming a stable solid–liquid dual-phase lubricating film, which effectively suppresses both adhesive wear and abrasive wear.

Although this study validated the effectiveness of the CaF_2_/[BMIM]PF_6_@PPSU system on YG8 cemented carbide tools, the underlying methodology is inherently versatile. The technical route of fabricating microtextures by laser processing followed by filling with multifunctional microcapsules can be readily extended to other advanced cutting tool materials, such as microtextured ceramic tools (Al_2_O_3_- and Si_3_N_4_-based) and ultra-hard tool materials (e.g., PCBN or PCD). Future research will focus on the lubrication performance of the microcapsule-resin composite within the microtextures on different tool substrates, aiming to further expand the industrial application range of self-lubricating tool technology.

## Figures and Tables

**Table 1:** Cutting Data at Different Depths of Cut.

No.	*v* (m/min)	*f* (mm/rev)	*a_p_* (mm)	*F_z_* (N)	*F_x_* (N)	*F_y_* (N)	*T* (°C)	*R_a_* (μm)
1	150	0.101	0.2	100.3	32.4	107.7	321.4	1.783
2	150	0.101	0.3	129.1	36.5	113.7	367.1	1.625
3	150	0.101	0.4	164.6	41.2	143.9	403.5	1.551 (min)
4	150	0.101	0.5	202.2	47.4	188.3	518.2	1.686

**Table 2:** Cutting Data at Different Cutting Speeds.

No.	*v* (m/min)	*f* (mm/rev)	*a_p_* (mm)	*F_z_* (N)	*F_x_* (N)	*F_y_* (N)	*T* (°C)	*R_a_* (μm)
1	100	0.101	0.4	149.1	37.3	127.5	397.4	1.984
2	150	0.101	0.4	164.4	41.2	143.9	403.5	1.551
3	200	0.101	0.4	152.7	38.8	134.8	422.1	1.427 (min)
4	250	0.101	0.4	176.9	44.6	151.4	458.3	1.56

## Data Availability

Data are contained within the article. The data presented in this study can be requested from the authors.
